# Influence of plant growth-promoting endophytes *Colletotrichum siamense* and *Diaporthe masirevici* on tomato plants (*Lycopersicon esculentum* Mill.)

**DOI:** 10.1080/21501203.2022.2050825

**Published:** 2022-03-17

**Authors:** Shalene da Silva da Silva Santos, Angela Aparecida da da Silva, Julio Cesar Polonio, Andressa Domingos Polli, Ravely Casarotti Orlandelli, João Arthur Dos Santos dos Santos Oliveira, José Usan Torres Brandão Filho, João Lúcio Azevedo, João Alencar Pamphile

**Affiliations:** a Department of Biotechnology, Genetics and Cell Biology, Universidade Estadual de Maringá, Maringá, Brazil; b Department of Agronomy, Universidade Estadual de Maringá, Maringá, Brazil; c Department of Genetics, College of Agriculture Luiz de Queiroz, Universidade de São Paulo, Piracicaba, Brazil

**Keywords:** Endophytes, phosphate solubilisation, IAA production, SPAD index, *fusarium oxysporum*

## Abstract

The protective and growth-promoting activities of *Colletrotrichum* and *Diaporthe* endophytes on tomato plants (*Lycopersicon esculentum* Mill.) are underexplored. We screened 40 endophytic fungi associated with Mexican shrimp plant (*Justicia brandegeana*) using an *in vitro* dual culture assay for *Fusarium oxysporum*, one of the most important phytopathogens of tomato plants. The three best antagonists, *Colletotrichum siamense* (JB224.g1), *C. siamense* (JB252.g1), and *Diaporthe masirevicii* (JB270), were identified based on multilocus sequence analysis. They were assessed *in vitro* for their inhibition of *F. oxysporum* and phosphate solubilisation capacity, and for the production of indole acetic acid. Greenhouse experiments verified the growth-promoting effects of these endophytes and the suppression of *F. oxysporum* symptoms in tomato plants.   Under greenhouse conditions, the JB252.g1 and JB270 isolates showed positive results for seedling emergence speed. The radicular system depth of plants inoculated with JB270 was greater than that in uninoculated plants (27.21 vs 21.95 cm). The soil plant analysis development chlorophyll metre (SPAD) index showed statistically significant results, especially for the endophyte JB224.g1 (36.99) compared to the control plants (30.90) and plants infected solely with *F. oxysporum* (33.64).

## Introduction

The fruit of the tomato plant (*Lycopersicon esculentum* Mill.) is one of the most consumed fruits worldwide. In 2019, globally the harvested area was approximately 5 million hectares (ha) and production estimated at 181 million tons. In 2019 in Brazil, tomato crops occupied approximately 55 thousand ha with an estimated production of 3.9 million tons (FAO Food and Agriculture Organization of the United Nations, 2020).

Tomato crops are highly susceptible to the attack of the fungus *Fusarium oxysporum*, the causal agent of the Wilt disease. The fungal growth occurs more commonly at higher temperatures, as well as in acidic and sandy soils. The most visible symptom, especially at the start of fruiting, is the yellowing of older leaves, which progresses to newer leaves. This is followed by the wilting of leaves during the hottest hours of the day. In addition, *Fusarium* wilt symptoms can have a one-sided appearance, corresponding to the side where the vascular infection initially occurred. Furthermore, darkening of the infected vascular tissues occurs and becomes more intense at the stem base. The initial stages of growth of infected tomato plants can be slow (McGovern 2015). The control of this pathogen is quite difficult because of its persistence in soil and its wide host range. Some chemicals are effective, but they are expensive and not environmentally friendly. Hence, alternative measures need to be identified and tested (Abdel-Monaim et al. 2011).

To mitigate these impacts, beneficial microorganisms with protective and growth-promoting effects are desired. Endophytic fungi are microbes that live in the interior of plants for part or all their life cycle, without harm to their hosts. They have several beneficial roles in plants, including protection against diseases by competing with phytopathogens for colonisation sites and nutrients, and production of antibiotics (Pamphile and Azevedo 2002; Khan et al. 2012; Khan and Lee 2013).

Plant growth promotion through the synthesis of phytohormones and/or increased tolerance to abiotic stresses are important fungal activities that favour the host (Mwangi et al. 2011; Yadav et al. 2011; Rashid et al. 2012; Mercado-Blanco and Lugtenberg 2014; Hamayun et al. 2017; Bilal et al. 2018; Ripa et al. 2019; Bouzouina, Kouadria and Lotmani 2021; Khalil et al. 2021; Ribeiro et al. 2021; Syamsia et al. 2021).

The growth-promoting and protective effects of *Colletotrichum* and *Diaporthe* endophytes on tomato plants have not been described. Colonisation of tomato plants by a *Colletotrichum* strain and its growth-promoting effects in greenhouse and open-field experiments has been previously described only in one study (Díaz-González et al. 2020). Seven days after inoculation with *Colletotrichum tofieldiae*, these authors observed that the tomato seedlings had shoots and roots significantly longer and had greater fresh weight compared with untreated plants. In addition, a significant increase in the number of buds (up to 64%), open flowers (up to 54%), and dry root weight were 2% higher than in control plants. Other studies have reported the effects of *Colletrotrichum* on different model plants, such as *Phaseolus vulgaris* (Oliveira et al. 2020) and *Arabidopsis thaliana* (Hiruma et al. 2016).

There are no descriptions in the literature of *Diaporthe* strains inoculated in tomato plants, and little is known about *in vivo* plant–*Diaporthe* interactions. Recently, Aldana et al. (2021) reported that a *Diaporthe* sp. endophytic strain caused a > 30% increase in both root and shoot biomass of tritordeum, a hybrid grain cereal developed from a cross between durum wheat (*Triticum durum*) and *Hordeum chilense*, a wild barley native to Chile and Argentina. The authors also described increased concentrations of calcium, magnesium, sulphur, iron, and boron in treated plants.

In the current study, two isolates of *C. siamense* (JB224.g1 and JB252.g1) and an isolate of *Diaporthe masirevicii* (JB270) appeared to be the most promising antagonists to *F. oxysporum* in a preliminary *in vitro* screening assay. To contribute to an in-depth understanding of the growth-promoting and protective activity of *Colletrotrichum* and *Diaporthe* strains on tomato plants, we investigated the protective action of these three endophytes on *Fusarium* wilt and their growth promotion effects on shoot height, radicular system depth, number of leaves, and soil plant analysis development chlorophyll metre (SPAD) index, and on the fresh and dry mass of the aerial part and roots of tomato plants.

## Materials and methods

### Fungal strains

Forty fungi isolated as endophytes from healthy leaves of Mexican shrimp plant (*Justicia brandegeana*) (Silva et al. 2020) were retrieved from the Collection of Endophytic and Environmental Microorganisms (CMEA) from the Laboratory of Microbial Biotechnology, Universidade Estadual de Maringá, Paraná, Brazil. *F. oxysporum* was also obtained from CMEA. Fungi were cultured on potato dextrose agar (PDA) at 28°C for 7 days before each experiment.

### Screening of endophytic fungi with F. oxysporum inhibitory activity in dual culture

A modified version of the dual culture method of Campanile et al. (2007) was used. Plugs (6 mm) from 7-day-old cultures of endophytes and phytopathogens were combined in triplicate and inoculated at 2.5 cm on opposite sides of 9-cm PDA dishes. As the control, a *F. oxysporum* plug was inoculated on one side of the dish. Dishes were randomly distributed in an incubator chamber at 28°C. After 7 days, the results were recorded using ImageJ software. The inhibition index percentage of mycelial growth (Im%) was calculated as 100 – (MT/MC) × 100, where MT is the mean of the triplicate area measured for treatment in cm^2^ and MC is the mean of the triplicate control area in cm^2^.

The competitive interactions between endophytes and *F. oxysporum* were determined according to the Badalyan Rating Scale (Badalyan et al. 2002). The three best antagonists were selected for molecular identification and evaluation of the antifungal activity of metabolic extracts, evaluation of phosphate solubilisation, evaluation of mycelial growth under different pH conditions, production of indole acetic acid (IAA), and *in vivo* tests in the greenhouse.

### Taxonomic identification of endophytes based on multilocus sequence analysis (MLSA)

Genomic DNA was extracted using the Power Soil DNA Isolation kit (MoBio Laboratories, USA) according to the manufacturer’s instructions. The endophytes were previously grown in petri dishes containing PDA, with 200 mg of mycelia used for the extraction. DNA integrity was checked by 1% agarose gel electrophoresis. For MLSA, partial sequences of the internal transcribed sequence ITS1-5.8S-ITS2 (ITS), translation elongation factor 1-α (EF1α), β-tubulin (TUB), and glyceraldehyde-3-phosphate dehydrogenase (GPDH) were used. For *Diaporthe* endophyte, ITS and EF1α sequences were used for typing. ITS, TUB, and GPDH were used for *Colletotrichum* strains. Primers used for amplification and PCR conditions are listed in Supplementary Table S1. PCR products were purified using shrimp alkaline phosphatase and exonuclease I (Sigma-Aldrich, USA). Samples were sequenced using the ABI-PRISM 3100 Genetic Analyser (Applied Biosystems, USA) by ACTGene Análises Moleculares (Brazil).

The sequences were treated and aligned using Geneious Prime v. 2019.1.1. Once the sequences showed high similarities in GenBank with other fungi, a phylogenetic analysis was performed based on pre-established data (Supplemental Tables S2 and S3). Sequences were then rescued and aligned using MAFFT (Katoh, Rozewicki and Yamada 2019). After alignment, multigene assembly of the sequences was performed. For phylogenetic analysis based on the maximum likelihood and Bayesian inference, MrModelTest v. 2.3, (Nylander 2004) was used to choose the best evolutionary model. The phylogenetic tree was constructed using MrBayes v. 2.2.4, (Ronquist et al. 2012), taking into consideration the parameters generated by MrModelTest, with Markov chain Monte Carlo, which lasted until the average standard deviation of the split frequencies was <0.01 (1.000.000. Bayesian probability was demonstrated on the nodes between each individual. The tree was edited using FigTree v. 1.4.2 (Rambaut 2009).

### In vitro F. oxysporum inhibitory activity of crude metabolic extracts produced by endophytes

To obtain the crude ethyl acetate extracts (CEAEs) of secondary metabolites, endophytes were inoculated into 500 mL Erlenmeyer flasks containing 250 mL of potato dextrose broth (PDB) and incubated in the dark at 28°C under stationary conditions for 21 days. The broth cultures were then filtered with sterile gauze to separate the fungal mycelia that were discarded. The cell-free media was centrifuged at 2750 × *g* for 15 min to separate the cellular debris. The liquid-liquid extraction of the supernatant and the recovery of the crude extract were performed as previously described (Orlandelli et al. 2012).

For the antifungal activity assay, 6-mm plugs from 7-day-old cultures of in vitro *F. oxysporum* cultures were combined with 6 mm paper plugs (sterile Whatman No. 4 filter paper) were combined in triplicate and inoculated at 4 cm on opposite sides of 9-cm PDA dishes. Paper plugs were inoculated with either 10 µL of 10 mg mL^−1^ CEAEs diluted in methanol (treatments), methanol only (negative control), or the broad-spectrum fungicide Benlate (Benomyl) (positive control). The dishes were then incubated at 28°C for 7 days, and pathogen growth in controls and treatments was compared. The inhibition index (lrb%) was calculated as previously described for the dual culture assay.

### Evaluation of phosphate solubilisation capacity

Modified Pikovskaya agar medium was used to determine the phosphate solubilisation capacity (Nopparat et al. 2009). The base solution (BS) was composed of (NH_4_)_2_SO_4_ (0.5 g), KCl (0.2 g), MgSO_4_.H_2_O (0.1 g), MnSO_4_.H_2_O (0.004 g), FeSO_4_.7H_2_O (0.002 g), NaCl (0.2 g), D-glucose (10 g, Sigma-Aldrich), yeast extract (0.5 g, Kasvi, Brazil), bacteriological agar (18 g, Acumedia, USA), and distilled water (900 mL). The pH was adjusted to 6.8. The first phosphate solution (PS1) was composed of xanthan gum (0.5 g, Sigma-Aldrich), Ca_3_(PO_4_)_2_ (0.5 g, β-tricalcium phosphate, Sigma-Aldrich) and distilled water. For the second solution (PS2), calcium phosphate was replaced with bone meal (0.5 g). All solutions were autoclaved (15 min at 121°C), mixed (BS+ PS1 and BS + PS2; pH adjusted to 6.6), and transferred to petri dishes.

To evaluate the growth of endophytes under different phosphorous sources, 6-mm plugs of each fungus were inoculated in petri dishes with BS+PS1 and BS+PS2 and incubated at 28°C for 7 days. The diameter of the halo surrounding the fungal colony and the colony diameter were measured daily using a graduated ruler (cm). The relative solubilisation efficiency was calculated as the diameter of solubilisation halo/diameter of colony × 100, using the mean of five replicates.

### Evaluation of effects of pH on mycelial growth

Plugs (6 mm) from 7-day-old cultures of endophytes were transferred to PDA dishes adjusted to different pH values (4, 5, 7, and 8). Additionally, mycelial plugs were transferred to PDA dishes supplemented with bromothymol blue and adjusted to pH 6.8. The experiment was conducted in a randomised block design with three replicates. Petri dishes were incubated at 28°C for up to 7 days. On day 6, mycelial growth was measured using ImageJ software.

### IAA production by endophytes

Endophytes were cultivated in PDB (10% w/v) containing 0.5 mM L-tryptophan for 7 days at 28°C in darkness in an orbital shaker-incubator at 110 rpm. The cultures were centrifuged at 15,000 × *g* for 5 min, and 1 mL of the supernatant was collected and added to the Salkowski reagent (2 mL). The samples were kept in the dark at room temperature for 30 min. Absorbance was read at 520 nm using a spectrophotometer. The amount of IAA was calculated from the standard curve prepared earlier with 5, 10, 25, 50, 75, and 100 μg mL^−1^ of IAA (Sigma-Aldrich).

### Evaluation of growth-promoting effects and Fusarium inhibitory activity of endophytes on tomato plants

The *in vivo* assays were performed in the greenhouse of the Agronomy Department, Universidade Estadual de Maringá, Brazil (23°24ʹ12.18″S and 51°56ʹ30.54″ O). Seeds were disinfected with alcohol 70% for 2 min and with sodium hypochlorite for 3 min, and then rinsed in sterile distilled water. This procedure was repeated 10 times. Two substrates were used for sowing the tomato seeds. Mecplant® substrate was composed of Pinus bark, vermiculite, acidity regulator, macro-and micronutrients. It was autoclaved at 121°C for 60 min. Soil was collected at the Universidade Estadual de Maringá and was previously autoclaved at 121°C for 120 min. After sterilisation, the soil was homogenised, and a sample (500 g) was collected for chemical and physical analyses. Granulometric analysis determined the content of coarse sand (43.7%), fine sand (4.8%), silt (12.5%), and clay (39.0%). Complete analyses of the chemical attributes are presented in the Supplementary Material (Table S6).

To obtain the mycelial suspension of endophytes and *F. oxysporum*, fungi were grown on petri dishes containing PDA at 28°C for 7 and 15 days, respectively. Then, 6-mm mycelial plugs were macerated in microtubes containing autoclaved distilled water (1.5 mL), which were shaken until complete homogenisation was achieved.

For the *in vivo* tests, an aliquot (1 mL) from the mycelial solution of each endophyte was inoculated into 300-mL polystyrene cups containing the Mecplant® substrate, which was then covered with plastic film and incubated at ambient temperature for 24 h. Each cup was inoculated with one tomato seed (at the same point of fungal inoculation) and kept under greenhouse conditions. Twenty-four days after sowing, the root system of the tomato seedlings was washed in running water and transplanted into 500-mL polystyrene cups containing a mixture (2:1 v/v) of soil and the Mecplant® substrate. Ten aliquots (1 mL) from the mycelial solution of each endophyte were individually inoculated into 10 cups. During the transplant of seedlings, some samples (inoculated and uninoculated with endophytes) were randomly selected for the *in vivo* antagonism test. They were infected with *F. oxysporum* suspension (1 mL) by inoculation near the roots. Ten replicates were used.

As a control, tomato seeds were sown in Mecplant® substrate uninoculated with endophytes, and seedlings were transplanted into 500-mL polystyrene cups without *F. oxysporum*. The controls used were the same for both the assays. All treatments and controls were maintained under greenhouse conditions, with an average temperature of 25–35°C. The experimental design was completely randomised, and replicates were composed of one vase with one plant, totalling 10 plants per treatment.

### Assessment of germination percentage, speed of seedling emergence, and final pH

Each treatment was evaluated in terms of germination percentage, seedling emergence, and final pH. On day 6, the first germination count was determined by counting the number of normal seedlings. After 7 days, the final germination count was obtained, and the results were expressed as the germination percentage (Brazil 2009). The emergence speed index (ESI) was calculated using the following formula: ESI = (E1/N1) + (E2/N2) + … (En/Nn), where ESI is the emergence speed index, En (E1, E2 …) is the number of seeds that had emerged, and Nn (N1, N2 …) is the number of days after sowing, from the first to the last count (Maguire 1962).

The final pH of each sample was measured according to EMBRAPA (Empresa Brasileira de Pesquisa Agropecuária) (1997), where 10 cm^3^ of soil was placed in a 100 mL Erlenmeyer flask and 25 mL of distilled water was added. The solution was stirred with a glass rod and allowed to stand for 1 h. The pH was then measured using a pH metre.

### Measurement of disease severity of the tomato plants infected with F. oxysporum

Disease severity was assessed 32 days after the inoculation of *F. oxysporum* using the ordinal scale (1 to 5) developed by Santos (1997). In the scale, 1 = plant free of symptoms; 2 = plant without wilt symptoms but presenting conspicuous vascular browning; 3 = plants showing vascular browning symptoms and wilt symptoms but without leaf yellowing; 4 = severe wilting associated with the presence of foliar necrosis and chlorosis; and 5 = dead plant.

### Evaluation of biometric parameters

The biometric parameters of shoot height (SH), radicular system depth (RSD), number of leaves (NL), and SPAD index, fresh and dry mass of the aerial part and roots (FM_AP_, DM_AP_, FM_R_, and DM_R_, respectively) were evaluated for the previously described treatments.

The SH of each plant was measured (cm) using a graduated ruler from the base of the shoot to the apical bud. Chlorophyll content was determined using a SPAD-502 chlorophyll metre (Spectrum Technologies, Inc., USA). The mean of each treatment was determined from the values obtained for the three leaves. For each leaf, readings were made at three separate points (two leaflets on opposite sides and the terminal central leaflet), and the mean was calculated using the SPAD metre.

To obtain the FM, the plants were collected using the destructive method, the leaves and roots were separated into identified plastic bags, and the mass of the leaves was measured with a precision balance. After weighing, the leaves in the identified paper bags were kept in a forced air circulation oven at 60°C for 72 h. The material was weighed again to obtain DM. The results were compared using the statistical analyses outlined below.

### Statistical analyses

The dual culture assay was performed in triplicate, and the means were analysed by analysis of variance (ANOVA) compared with the Scott-Knott test (p < 0.05). The antifungal activity of the crude extract of secondary metabolites produced by the endophytes was determined in triplicate and the solubilisation capacity determined using five replicates were analysed by ANOVA. Means were compared by the Tukey test (p < 0.05). All statistical analyses were carried out using the Sisvar software 4.3 (Ferreira 2011).

The greenhouse experiments were performed with ten replicates. Biometric parameters were analysed using two-way ANOVA aiming to evaluate the effects of the fungi in plants inoculated with phytopathogens and plants with endophytes.

## Results

### Multigene molecular identification of endophytes selected in dual culture test

**Figure 1.**
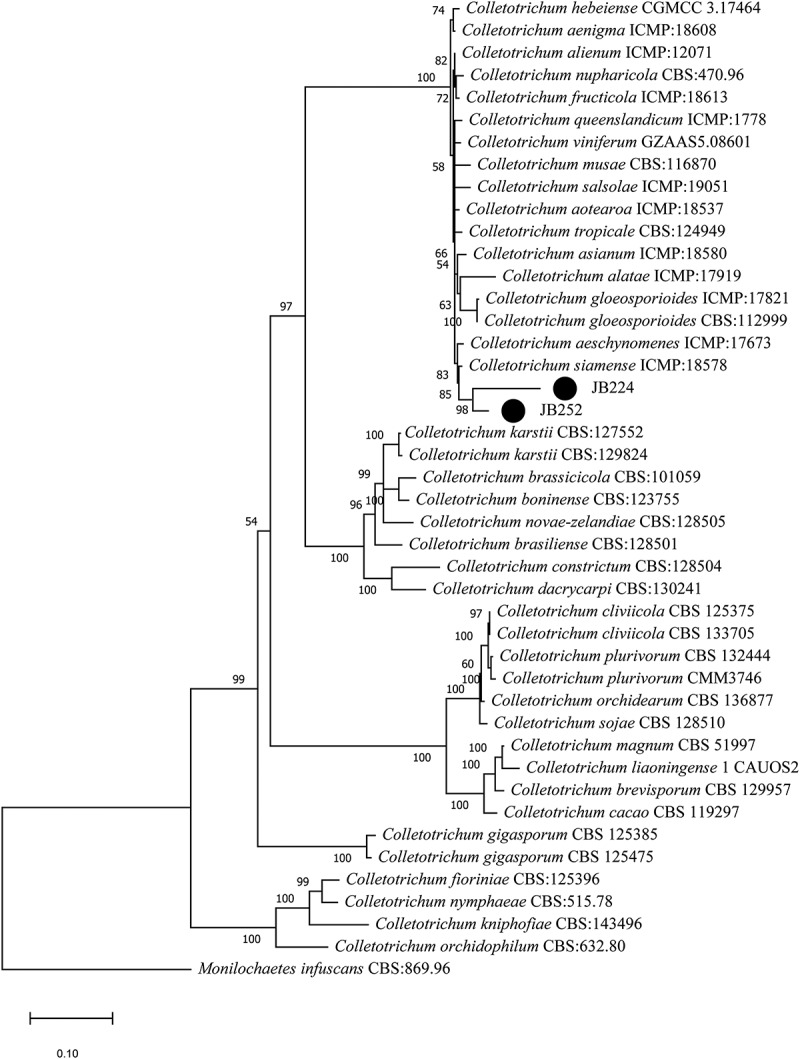
Phylogenetic tree of *Colletotrichum* endophytes from *Justicia brandegeana* with other fungi obtained from the GenBank database. The tree was constructed using the neighbour-joining method and p-distance for nucleotides with the pairwise gap deletion in the software MEGA version 6.05. The number on the tree branches represents the number of times (as a percentage) the group on the right occurred on the same node during the consensus evaluation (bootstrapped with 10,000 replicates). Endophytic isolates are indicated by black circles (●). Sequences from *Monilochaetes infuscans* CBS:869.96 were used as outgroup.

**Figure 2.**
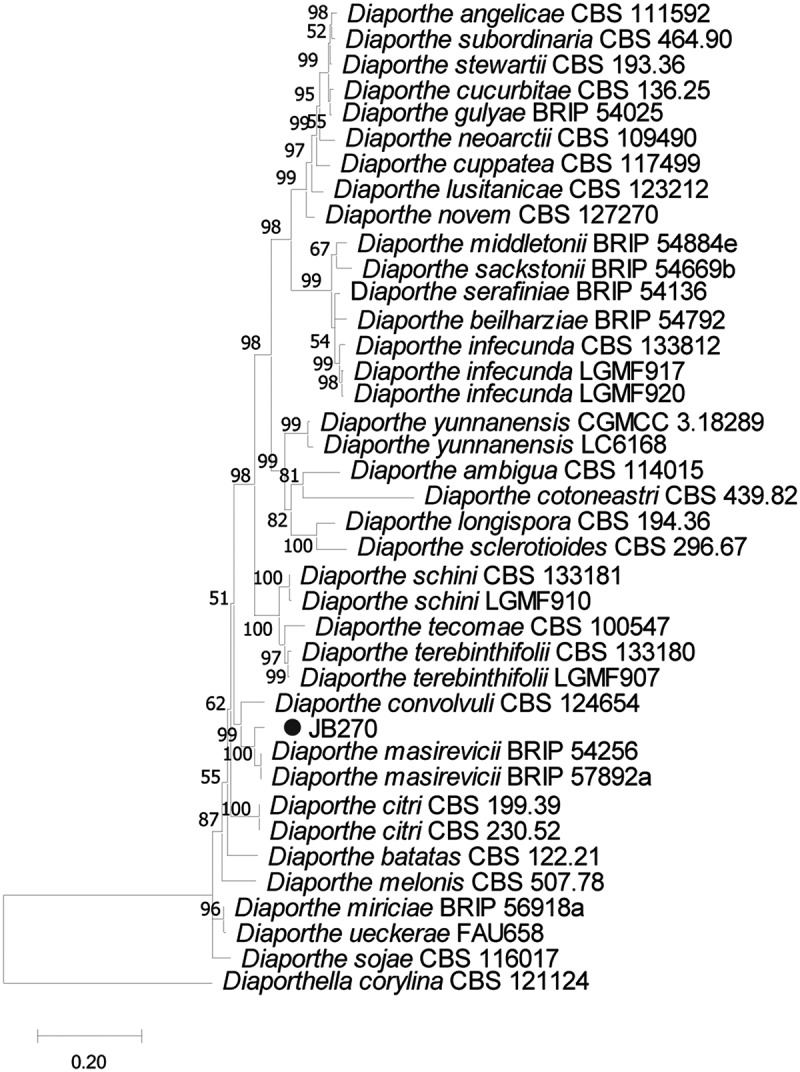
Phylogenetic tree of the *Diaporthe* endophytic isolate from *Justicia brandegeana* with other fungi obtained from the GenBank database. The tree was constructed using the neighbour-joining method and p-distance for nucleotides with the pairwise gap deletion in the software MEGA version 6.05. The number on the tree branches represents the number of times (as a percentage) the group on the right occurred on the same node during the consensus evaluation (bootstrapped with 10,000 replicates). Endophytic isolate is indicated by a black circle (●). Sequences from *Diaporthella corylina* CBS 121124 were used as outgroup.

The sequencing data from the three endophytes selected by the dual culture test (described below) were initially compared with other GenBank sequences. The results (Table S4, Supplementary Material) showed a higher identity of JB224.g1 and JB252.g1 to *Colletotrichum* species, whereas JB70 was closely related to *Diaporthe* strains. The MLSA phylogenetic analyses using the ITS, TUB, and GPDH partial sequences revealed that the endophytic strains JB224.g1 and JB252.g1 belonged to the *Colletotrichum gloeosporioides* complex, showing higher genetic similarities with *C. siamense* (Figure 1, Table S4). Analysis of ITS and EF1α from JB270 showed that this isolate was closely related to other *Diaporthe masirevicii* strains (Figure 2, Table S4).

### In vitro anti-Fusarium activity of endophytes and their crude metabolic extracts

Endophytes with *anti-F. oxysporum* activity was screened using a dual culture assay. According to the Scott-Knott test, results were distributed into six statistical groups, and details regarding the inhibition index and endophyte-pathogen interactions are shown in Table S5 (Supplementary Material). The aforementioned results highlighted the isolates JB224.g1, JB252.g1, and JB270 as more promising antagonists, with inhibition rates varying between 68.19 and 54.21%. Endophytes JB224.g1 and JB252.g1 presented deadlock interaction with mycelial contact (competitive interaction type A) and deadlock interaction at a distance (type B), respectively. Endophyte JB270 partially overgrew the phytopathogen (interaction C, subtype C_A1_).

These three fungi were selected for further analysis of the antifungal activity of their CEAEs. As shown in Table 1, all CEAEs presented anti-*F. oxysporum* activity, but only the treatment with CEAE_JB270_ was statistically different in comparison to the negative control (methanol).

**Table 1.** Taxonomic identification based on sequencing of the ITS1-5.8S-ITS2 region of the rDNA.

Endophyte	Taxonomic identification*	Genbank database accession no.	Identity
JB224.g1	*Colletotrichum siamense*	*C. siamense* (KP703350.1)	98%
JB252.g1	*Colletotrichum siamense*	*C. siamense* (KP703350.1)	99%
JB270	*Diaporthe masirevicii*	*D. masirevicii* (KJ197281.1)	98%

*Based on a BLASTn search and phylogenetic analyses. **Accession no. with highest identity on Genbank database.

### Phosphate solubilisation, growth under different pH values, and IAA production

The three endophytes showed a capacity for phosphate solubilisation 2 days after inoculation as evident by the formation of a halo around the colonies. However, in the presence of the bone meal substrate, the isolates did not show any halo formation, indicating that this source of phosphorus could not be solubilised by the endophytes. On day 4 of growth, the relative solubilisation efficiencies of JB224.g1 and JB270 were 166% and 109%, respectively, while the endophyte JB252.g1 presented a higher efficiency (184%) on day 3 of growth. The collective results indicate that the area of solubilisation was approximately two times larger than the fungal size for the isolates JB224g.1 and JB252g.1 Data on mycelial growth, solubilisation halo, and relative solubilisation efficiency of endophytes are detailed in Table 2.

**Table 2.** Inhibition index (Im%) and competitive interaction between the 40 endophytic fungal isolates and the phytopathogen *F. oxysporum* (FO).

Fungal isolates	Mycelial growth of FO (cm)*	Im%	Interaction**
JB252.g1	13.42^a^ ± 0.23	68.19	B
JB270	16.10^a^ ±7.95	61.84	C_A1_
JB224.g1	19.32^b^ ± 3.73	54.21	A
JB09	19.50^b^ ± 1.94	53.76	C_A1_
JB07	20.94^c^ ± 5.38	50.35	B
JB176	21.07^c^ ± 1.20	50.05	B
JB22	21.61^c^ ± 0.45	48.77	B
JB200	21.84^c^ ± 2.04	48.22	C_A1_
JB202	21.93^c^ ± 1.18	48.01	C_A1_
JB123.g1	22.15^c^ ± 0.29	47.49	A
JB35	22.35^c^ ± 0.19	47.01	B
JB44.g1	22.62^c^ ± 4.17	46.38	A
JB72	22.70^c^ ± 1.97	46.19	A
JB185	22.82^c^ ± 2.10	45.91	A
JB196	22.82^c^ ± 4.86	45.90	A
JB57	23.33^c^ ± 1.39	44.70	C_A1_
JB261	23.48^c^ ± 2.85	44.34	A
JB89	23.65^c^ ± 1.15	43.94	A
JB55	23.76^c^ ± 1.11	43.67	C_A1_
JB27. g1	23.92^c^ ± 1.21	43.29	A
JB162	25.41^c^ ± 0.95	39.77	A
JB76	25.64^c^ ± 2.29	39.22	A
JB100	26.14^c^ ± 1.41	38.02	A
JB10	26.15^c^ ± 3.82	38.01	A
JB90	26.16^c^ ± 1.34	37.99	A
JB101	26.54^c^ ± 0.78	37.09	A
JB122	26.56^c^ ± 0.79	37.04	A
JB73	26.70^c^ ± 1.22	36.70	A
JB118	26.93^c^ ± 0.27	36.15	A
JB207.g1	27.08^c^ ± 2.62	35.81	A
JB175	28.38^d^ ± 1.36	32.71	A
JB99	29.29^d^ ± 2.52	30.56	A
JB301.g1	29.60^d^ ± 3.42	29.82	A
JB107	30.14^d^ ± 1.60	28.55	A
JB268	30.23^d^ ± 2.16	28.33	A
JB150	30.24^d^ ± 1.48	28.32	A
JB25	30.29^d^ ± 3.74	28.20	A
JB206	31.37^d^ ± 1.99	25.63	A
JB173	35.47^e^ ± 0.49	15.92	A
FO (control)	42.18 ^f^ ± 1.99	-	-

*Mean of triplicates followed by different letters indicates that the values are significantly different according to the Scott-Knott test (p < 0.05). **Badalyan rating scale (Badalyan et al. 2002) where A = deadlock with mycelial contact, B = deadlock at a distance, and CA1 = partial replacement after initial deadlock with mycelial contact.

In addition, isolate JB224.g1 showed higher *in vitro* growth at pH values of 5 and 7. ANOVA indicated that the isolates JB252.g1 and JB270.g1 showed no significant difference across the pH values evaluated in this study, suggesting that they did not show pH-responsive behaviour (Table 3).

**Table 3.** *In vitro* antifungal activity of the CEAEs produced by the three selected fungal endophytes (identified as JB224.g1, JB252.g1, and JB270) against *F. oxysporum* (FO).

Trataments	Mycelial growth of FO (cm)*	Im%
CEAE_JB224.g1_	39.65^bc^ ± 1.48	9.1
CEAE_JB252.g1_	40.68^bc^ ± 1.65	6.7
CEAE_JB270_	37.61^b^ ± 1.40	13.8
Methanol (negative control)	43.61^c^ ± 2.28	-
Benlate (positive control)	31.87^a^ ± 2.19	26.9
CV (%)	4.42	-

*Mean of triplicates followed by the same letters did not differ according to the Tukey test (p < 0.05). CV is the coefficient of variation.

When endophytes were inoculated in culture medium with bromothymol blue, only isolate JB270 was able to change the colour to yellow, indicating acidification of the medium. The culture medium remained blue for the other two endophytes, indicating a basic pH. The three endophytes tested were IAA-positive when cultured in PDB supplemented with L-tryptophan. The amount of IAA for each endophyte, calculated using the standard curve (R^2^ = 0.9882), was 290 µg mL^−1^ for JB224.g1, 40.2 µg mL^−1^ for JB252.g1, and 0.49 µg mL^−1^ for JB270.

### Growth-promoting effects of endophytes on tomato plants and wilt suppression

**Figure 3.**
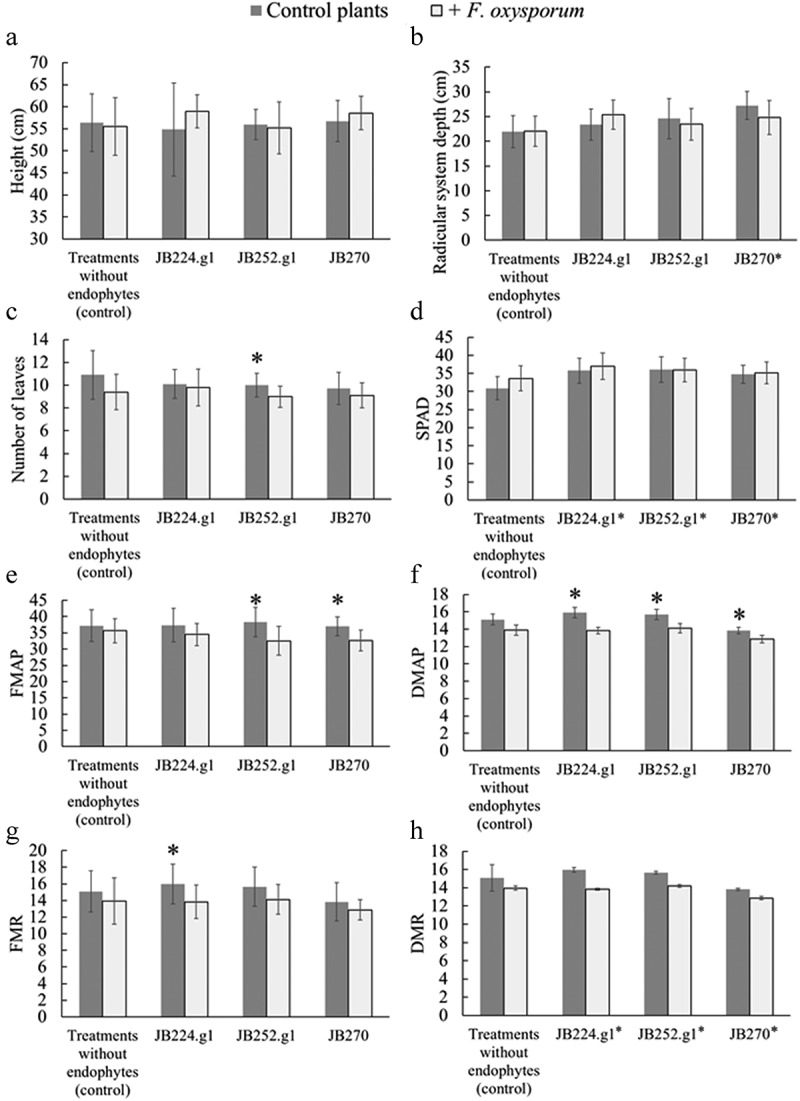
Graphs of plant fitness when inoculated with endophytes and phytopathogens: A) shoot height, B) radicular system depth, C) number of leaves, D) SPAD index: chlorophyll content, E) FMAP: fresh mass of aerial part, F) DMAP: dry mass of aerial part, G) FMR: fresh mass of roots, H) DMR: dry mass of roots. Endophytes = *C. siamense* (codes JB224.g1 and JB252.g1) and *D. masirevicii* (JB270). Asterisks (*) onto bars shows significant differences by two-way ANOVA (p < 0.05) between plants with or without *F. oxysporum* grouped by endophyte. Asterisks in horizontal legend shows that the endophyte group is more effective that plants without endophytes.

An overview of plant fitness is shown in Figure 3, and the values and two-way ANOVA F-values are detailed in the Supplementary Material (Table S7). The RSD of plants inoculated with JB270 was greater than that of uninoculated plants, with mean heights of 27.21 and 21.95 cm, respectively, indicating an interaction between the inoculated endophytic fungus and nutrient seeking by plants.

The NL in plants inoculated with JB252.g1 differed statistically from the treatments with these endophyte + *F. oxysporum* (Figure 3C). To SPAD index all treatments with endophytes showed statistically significant results with higher values (Figure 3D). These results of SPAD index demonstrate that the presence of endophytes could stimulate the photosynthetic rate of plants, regardless of the presence of the phytopathogen.

Regarding DM (Figures 3F and 3 H), a difference between the treatments with plants inoculated with the endophytes was observed to the tree strains. To DM_AP_ values were observed differences between treatments with endophytes + *F. oxysporum* and plants only inoculated with endophytic strains, showing that the phytopatogenic strain held their negative influence in apical biomass even with the presence of endophytes. In the DM_R_ analysis, the results indicating that the endophyte-plant interaction could influence the accumulation of roots dry biomass. However, the FM_AP_ and FM_R_ of endophyte-inoculated tomato plants presented different results. To FM_AP_ (Figure 3E) the endophytes JB252.g1 and JB270 showed higher values compared to plants inoculated with these strains and the phytopathogen. To FM_R_ the endophyte JB224.g1 demonstrated a better result compared with the treatment with co-inoculation with phytopathogen. So, these results to DM and FM demonstrated that the endophytes present plant growth promoting activity to these features, but, do not showed the same efficacy when the plant is contaminated with *F. oxysporum*.

**Figure 4.**
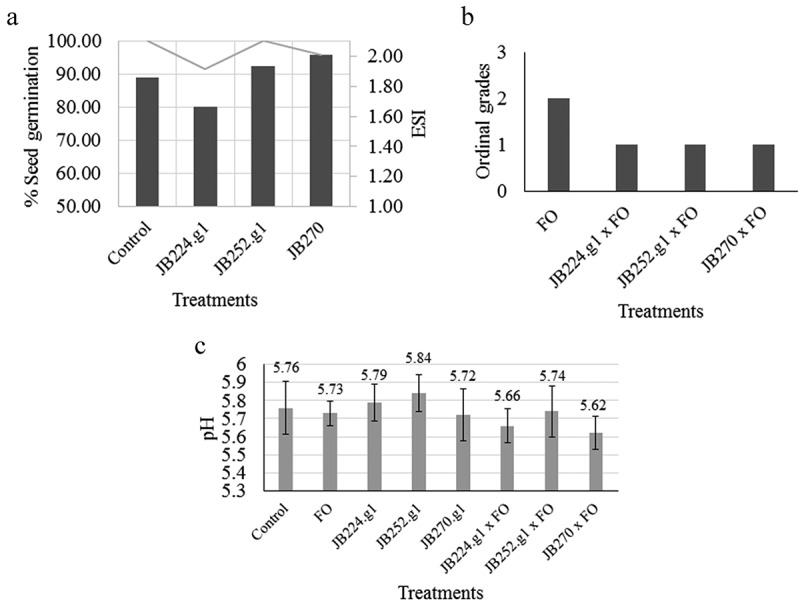
A) Columns represent the emergence speed index (ESI) and the lines represent germination percentage of tomato seedlings after treatment with fungal endophytes. B) Mean of ordinal grades attributed to the visible wilt symptoms caused by *F. oxysporum* (FO) in tomato plants. C) Mean of soil pH for each treatment after collection of plants. The bars indicate the standard deviation. Endophytes = *C. siamense* (codes JB224.g1 and JB252.g1) and *D. masirevicii* (JB270).

The endophytes JB252.g1 and JB270 showed positive results for seedling emergence speed (Figure 4A). No visible disease symptoms were observed during the *in vivo* tests, indicating that the endophytes did not cause any apparent deleterious effects on tomato plants. Therefore, the effectiveness of endophytes in suppressing *Fusarium* infection can be evaluated. In the control plants, the pathogen did not show dramatic virulence, causing only slight wilting in the plants. Analysis of the *in vivo* endophyte-pathogen interaction showed that slight wilt was not observed (Figure S1, Supplementary Material). These results confirmed that the plant-endophyte interaction promoted a decrease in the damage caused by *F. oxysporum* on tomato plants (Figure 4B).

Moreover, considering the evaluation of soil pH of the tomato plant before and after inoculation with the pathogen, no statistically significant differences were observed between the treatments and the control. This suggests that the interaction between the pathogen and endophytic fungi did not alter the pH of the tomato rhizosphere (Figure 4C).

## Discussion

Research concerning plant-microorganism interactions has progressed significantly in recent years. This is highlighted in the investigation of the plant microbiome linking microbial ecology and biological functioning of host plants, in addition to viewing microorganisms as a reservoir of additional genes and functions for their hosts (Vandenkoornhuyse et al. 2015). Nevertheless, even if this interaction initially appears to be symptomless, the additive ecological functions supported by the plant microbiome are acknowledged as a major trait extender of the ability of plants to adapt to many environmental conditions and changes (Bulgarelli et al. 2013).

Endophytism, defined as being asymptomatically established inside living plant tissue (Kusari and Spiteller 2012), is a unique cost-benefit association. Furthermore, fungal endophytes can be considered protective and/or growth-promoting agents, with some endophytes from traditional rice varieties having the capacity to significantly increase plant growth (Aly et al. 2011; Atugala and Deshappriya 2015).

In the present study, inoculation of the endophytic fungi *C. siamense* (strains JB224.g1 and JB252.g1) and *D. masirevicii* (JB270) in tomato plants increased the plant biomass. Previous research reports an increase in the biomass of tomato plants inoculated with *Trichoderma harzianum* and arbuscular mycorrhizal fungi (Mwangi et al. 2011). Furthermore, it has been shown previously that among the 28 *Trichoderma* isolates tested, 12 acted as growth promoters in tomato plants by allowing an increase in plant dry matter above 100% (Fontanelle et al. 2011).

Previously, the percentage of germination and vigour of seedlings when either the seeds of sunflowers were inoculated with *T. harzianum* or the soil was treated with *Penicillium chrysogenum* before sowing was investigated (Nagaraju et al. 2012; Murali et al. 2013). In both studies, the germination and vigour of the seedlings was higher than that of the control. It was also apparent that the endophyte *D. masirevicii* JB270 had the best germination speed index. However, these treated seeds did not achieve 100% germination.

Similarly, in this study, when endophytes (JB224.1, JB252.g1, and JB270) and *F. oxysporum* were inoculated together in tomato plants and kept under greenhouse conditions, wilt symptoms were not detected. In this study, plants inoculated with the pathogen *F. oxysporum* showed signs of wilt, which was not observed in control plants or plants inoculated with pathogen-endophyte combinations. This could indicate a positive plant-endophyte interaction that protected the plant against the pathogen. To understand this result, the photosynthetic capacity of the tomato plants was indirectly measured using a chlorophyll metre. Chlorophyll analysis showed that plants grown in soil inoculated with endophytes had higher SPAD indices than the control plants. This result is similar to previous research that investigated the effects of *Glomus versiforme* inoculum on watermelon grown under water stress and found that mycorrhizal colonisation enhanced the photosynthetic capacity and drought tolerance of plants (Mo et al. 2016).

In the current study, the three tested *J. brandegeana* endophytes (JB224.g1, JB252.g1, and JB270) were positive in the phosphate solubilisation assay on solid medium. Similarly, Hernandez-Leal et al. (2011) reported that *Paecilomyces lilacinus* was efficient for the *in vitro* solubilisation of phosphate and favoured the availability of phosphorus in soil. Additionally, Marra et al. (2012) demonstrated that microorganisms can use mechanisms such as the production and release of low molecular weight organic acids to solubilise precipitated forms of phosphorus, such as iron and aluminium phosphates in acidic soils and calcium phosphate in basic soils.

## Conclusions

In an attempt to minimise the use of chemical pesticides, biological control agents have emerged as an important tool in agricultural biotechnology (Gazis and Chaverri 2015; Suryanarayanan et al. 2018). Thus, *in vitro* tests (Rocha et al. 2011; Kumar and Kaushik 2013) and *in vivo* tests (Alwathnani and Perveen 2012) investigating the use of a fungal antagonist to suppress or eradicate pathogens should be performed in the future, especially in the search for novel species that can promote these protective effects. In this study, the endophytes tested showed *in vitro* inhibition of *F. oxysporum*. Furthermore, *in vivo* results showed a decrease in the symptoms of wilt disease in tomato plants. There could be several reasons, including the inhibition of *F. oxysporum*, reduction of the phytopathogen population by competition, or induction of plant resistance. Therefore, this study suggests that an increased photosynthetic capacity of endophyte-inoculated plants may have a possible connection with an increased plant resistance to pathogens such as *F. oxysporum*.

## Supplementary Material

Supplemental Material
