# Supplementary material for: Influence of plant growth-promoting endophytes Colletotrichum siamense and Diaporthe masirevici on tomato plants (Lycopersicon esculentum Mill.)
Source: Mycology. 2022 Mar 17;13(4):257–70. doi: 10.1080/21501203.2022.2050825 (PMC9673798; doi:10.1080/21501203.2022.2050825)
Supplement: Supplemental Material [file TMYC_A_2050825_SM0888.docx]

**SUPPLEMENTARY MATERIAL**

Influence of plant growth-promoting endophytes *Colletotrichum siamense* and *Diaporthe masirevici* on tomato plants (*Lycopersicon esculentum* Mill.)

Shalene da Silva Santos^a^, Angela Aparecida da Silva^a^, Julio Cesar Polonio^a*^, Andressa Domingos Polli^a^, Ravely Casarotti Orlandelli^a^, João Arthur dos Santos Oliveira^a^, José Usan Torres Brandão Filho^b^, João Lúcio Azevedo^c^, João Alencar Pamphile^a†^

*^a^Department of Biotechnology, Genetics and Cell Biology, Universidade Estadual de Maringá, 87020-900, Maringá, Paraná, Brazil*

*^b^Department of Agronomy, Universidade Estadual de Maringá, 87020-900, Maringá, Paraná, Brazil*

*^c^Department of Genetics, College of Agriculture Luiz de Queiroz, Universidade de São Paulo, 13418-900, Piracicaba, São Paulo, Brazil*

^†^*in memoriam*

***Corresponding author:

Julio Cesar Polonio

Avenida Colombo, 5.790, Jardim Universitário, 87020-900, Maringá, Paraná, Brazil

Phone: (55) 44 3011-4893. E-mail: jcpolonio2@uem.br

**Table S1.** Primers and PCR conditions.

| **Gene/ loci** | **Primers** | **Sequences (5’→3’)** | **PCR condition** | **References** |
| --- | --- | --- | --- | --- |
| ITS | ITS1  ITS4 | TCCGTAGGTGAACCTGCGG  TCCTCCGCTTATTGATATGC | 92 ºC: 4 min; (92º C 40s, 52 ºC, 1 min and 30 s, 72 ºC, 2 min) x 35 cycles; 72 ºC, 5 min | White et al. (1990) |
| EF1α | EF1-728F  EF1-986R | CATCGAGAAGTTCGAGAAGG  TACTTGAAGGAACCCTTACC | 94 ºC: 4 min; (94º C 1 min, 55 ºC, 1 min, 72 ºC, 1 min) x 40 cycles; 72 ºC, 3 min | Carbone and Kohn (1999) |
| TUB | T1  Bt-2b | AACATGCGTGAGATTGTAAGT  ACCCTCAGTGTAGTGACCCTTGGC | 94 ºC: 4 min; (94º C 1 min, 55 ºC, 1 min, 72 ºC, 1 min) x 40 cycles; 72 ºC, 3 min | Glass and Donaldson (1995), O’Donnell and Cigelnik (1997) |
| GPD | GPD1F  GPD2R | CAACGGCTTCGGTCGCATTG  GCCAAGCAGTTGGTTGTGC | 96 ºC: 2 min; (96 ºC 1 min, 48 ºC, 45 s, 72 ºC, 45 s) x 30 cycles, with addition of 4 s/ cycle in the extension step; 72 ºC, 10 min. | Berbee et al. (1999) |

**Table S2.** *Colletotrichum* sequences retrieved from the GenBank for phylogenetic analysis.

| **Fungal strains** | **GenBank accession number** | | | **References** |
| --- | --- | --- | --- | --- |
|  | **ITS** | **TUB** | **GPDH** |  |
| *Colletotrichum aenigma* ICMP:18608 | JX010244 | JX010389 | JX010044 | Fu et al. (2019) |
| *Colletotrichum aeschynomenes* ICMP:17673 | JX010176 | JX010392 | JX009930 | Fu et al. (2019) |
| *Colletotrichum alatae* ICMP:17919 | JX010190 | JX010383 | JX009990 | Fu et al. (2019) |
| *Colletotrichum alienum* ICMP:12071 | JX010251 | JX010411 | JX010028 | Fu et al. (2019) |
| *Colletotrichum aotearoa* ICMP:18537 | JX010205 | JX010420 | JX010005 | Fu et al. (2019) |
| *Colletotrichum asianum* ICMP:18580 | FJ972612 | JX010406 | JX010053 | Fu et al. (2019) |
| *Colletotrichum boninense* CBS:123755 | JQ005153 | JQ005588 | JQ005240 | Fu et al. (2019) |
| *Colletotrichum brasiliense* CBS:128501 | JQ005235 | JQ005669 | JQ005322 | Fu et al. (2019) |
| *Colletotrichum brassicicola* CBS:101059 | JQ005172 | JQ005606 | JQ005259 | Fu et al. (2019) |
| *Colletotrichum brevisporum* CBS 129957 | MG600762 | MG601029 | MG600822 | Damm et al. (2019) |
| *Colletotrichum cacao* CBS 119297 | MG600772 | MG601039 | MG600832 | Damm et al. (2019) |
| *Colletotrichum cliviicola* CBS 125375 | MG600733 | MG601000 | MG600795 | Damm et al. (2019) |
| *Colletotrichum cliviicola* CBS 133705 | MG600732 | MG600999 | MG600794 | Damm et al. (2019) |
| *Colletotrichum constrictum* CBS:128504 | JQ005238 | JQ005672 | JQ005325 | Fu et al. (2019) |
| *Colletotrichum dacrycarpi* CBS:130241 | JQ005236 | JQ005670 | JQ005323 | Fu et al. (2019) |
| *Colletotrichum fioriniae* CBS:125396 | JQ948299 | JQ949950 | JQ948629 | Fu et al. (2019) |
| *Colletotrichum fructicola* ICMP:18613 | JX010167 | JX010388 | JX009998 | Fu et al. (2019) |
| *Colletotrichum gigasporum* CBS 125475 | KF687732 | KF687872 | KF687835 | Liu et al. (2014) |
| *Colletotrichum gigasporum* CBS 125385 | KF687723 | KF687874 | KF687836 | Liu et al. (2014) |
| *Colletotrichum gloeosporioides* CBS:112999 | JQ005152 | JQ005587 | JQ005239 | Damm et al. (2019) |
| *Colletotrichum gloeosporioides* ICMP:17821 | JX010152 | JX010445 | JX010056 | Fu et al. (2019) |
| *Colletotrichum hebeiense* CGMCC 3.17464 | KF156863 | KF288975 | KF377495 | Fu et al. (2019) |
| *Colletotrichum karstii* CBS:127552 | JQ005217 | JQ005651 | JQ005304 | Damm et al. (2012) |
| *Colletotrichum karstii* CBS:129824 | JQ005215 | JQ005649 | JQ005302 | Damm et al. (2012) |
| *Colletotrichum kniphofiae* CBS:143496 | MH107884 | MH108037 | MH107998 | Crous et al. (2018) |
| *Colletotrichum liaoningense* CAUOS2 | KP890104 | KP890111 | KP890135 | Damm et al. (2019) |
| *Colletotrichum magnum* CBS 51997 | MG600769 | MG601036 | MG600829 | Damm et al. (2019) |
| *Colletotrichum musae* CBS:116870 | JX010146 | HQ596280 | JX010050 | Fu et al. (2019) |
| *Colletotrichum novae-zelandiae* CBS:128505 | JQ005228 | JQ005662 | JQ005315 | Fu et al. (2019) |
| *Colletotrichum nupharicola* CBS:470.96 | JX010187 | JX010398 | JX009972 | Fu et al. (2019) |
| *Colletotrichum nymphaeae* CBS:515.78 | JQ948197 | JQ949848 | JQ948527 | Fu et al. (2019) |
| *Colletotrichum orchidearum* CBS 136877 | MG600739 | MG601006 | MG600801 | Damm et al. (2019) |
| *Colletotrichum orchidophilum* CBS:632.80 | JQ948151 | JQ949802 | JQ948481 | Fu et al. 2019 |
| *Colletotrichum plurivorum* CBS 132444 | MG600720 | MG600987 | MG600783 | Damm et al. (2019) |
| *Colletotrichum plurivorum* CMM3746 | KC702981 | KC992328 | KC702942 | Damm et al. (2019) |
| *Colletotrichum queenslandicum* ICMP:1778 | JX010276 | JX010414 | JX009934 | Fu et al. (2019) |
| *Colletotrichum salsolae* ICMP:19051 | JX010242 | JX010403 | JX009916 | Fu et al. (2019) |
| *Colletotrichum siamense* ICMP:18578 | JX010171 | JX010404 | JX009924 | Fu et al. (2019) |
| *Colletotrichum sojae* CBS 128510 | MG600751 | MG601018 | MG600812 | Damm et al. (2019) |
| *Colletotrichum tropicale* CBS:124949 | JX010264 | JX010407 | JX010007 | Fu et al. (2019) |
| *Colletotrichum viniferum* GZAAS5.08601 | JN412804 | JN412813 | JN412798 | Fu et al. (2019) |
| *Monilochaetes infuscans* CBS:869.96* | JQ005780 | JQ005864 | JX546612 | Liu et al. (2014) |

*outgroup

**Table S3.** *Diaporthe* sequences retrieved from the GenBank for phylogenetic analysis.

| **Fungal strains** | **GenBank accession number** | | | **References** |
| --- | --- | --- | --- | --- |
|  | **ITS** | **TEF** | |  |
| *Diaporthe ambigua* CBS 114015 | KC343010 | KC343736 | | Gomes et al. (2013) |
| *Diaporthe angelicae* CBS 111592 | KC343027 | KC343753 | Gomes et al. (2013) | |
| *Diaporthe batatas* CBS 122.21 | KC343040 | KC343766 | Gomes et al. (2013) | |
| *Diaporthe beilharziae* BRIP 54792 | JX862529 | X862535 | Thompson et al. (2015) | |
| *Diaporthe citri* CBS 199.39 | KC343051 | KC343777 | Gomes et al. (2013) | |
| *Diaporthe citri* CBS 230.52 | KC343052 | KC343778 | Gomes et al. (2013) | |
| *Diaporthe convolvuli* CBS 124654 | KC343054 | KC343780 | Gomes et al. (2013) | |
| *Diaporthe cucurbitae* CBS 136.25 | KC343031.1 | KC343757.1 | Gomes et al. (2013) | |
| *Diaporthe cuppatea* CBS 117499 | KC343057 | KC343783 | Gomes et al. (2013) | |
| *Diaporthe gulyae* BRIP 54025 | JF431299 | JN645803 | Thompson et al. (2015) | |
| *Diaporthe infecunda* LGMF917 | KC343129 | KC343855 | Gomes et al. (2013) | |
| *Diaporthe infecunda* LGMF920 | KC343131 | KC343857 | Gomes et al. (2013) | |
| *Diaporthe infecunda* CBS 133812 | KC343126 | KC343852 | Gomes et al. (2013) | |
| *Diaporthe longispora* CBS 194.36 | KC343135 | KC343861 | Gomes et al. (2013) | |
| *Diaporthe lusitanicae* CBS 123212 | KC343136 | KC343862 | Gomes et al. (2013) | |
| *Diaporthe masirevicii* BRIP 57892a | KJ197276 | KJ197238 | Thompson et al. (2015) | |
| *Diaporthe masirevicii* BRIP 54256 | KJ197277 | KJ197239 | Thompson et al. (2015) | |
| *Diaporthe melonis* CBS 507.78 | KC343142 | KC343868 | Gomes et al. (2013) | |
| *Diaporthe middletonii* BRIP 54884e | KJ197286 | KJ197248 | Thompson et al. (2015) | |
| *Diaporthe miriciae* BRIP 56918a | KJ197284 | KJ197246 | Thompson et al. (2015) | |
| *Diaporthe neoarctii* CBS 109490 | KC343145 | KC343871 | Gomes et al. (2013) | |
| *Diaporthe novem* CBS 127270 | KC343156 | KC343882 | Gomes et al. (2013) | |
| *Diaporthe sackstonii* BRIP 54669b | KJ197287 | KJ197249 | Thompson et al. (2015) | |
| *Diaporthe schini* CBS 133181 | KC343191 | KC343917 | Gomes et al. (2013) | |
| *Diaporthe schini* LGMF910 | KC343192 | KC343918 | Gomes et al. (2013) | |
| *Diaporthe sclerotioides* CBS 296.67 | KC343193 | KC343919 | Gomes et al. (2013) | |
| *Diaporthe serafiniae* BRIP 54136 | KJ197273 | KJ197235 | Thompson et al. (2015) | |
| *Diaporthe sojae* CBS 116017 | KC343197 | KC343923 | Gomes et al. (2013) | |
| *Diaporthe stewartii* CBS 193.36 | FJ889448 | GQ250324 | Thompson et al. (2011) | |
| *Diaporthe subordinaria* CBS 464.90 | KC343214 | KC343940 | Gomes et al. (2013) | |
| *Diaporthe tecomae* CBS 100547 | KC343215 | KC343941 | Gomes et al. (2013) | |
| *Diaporthe terebinthifolii* CBS 133180 | KC343216 | KC343942 | Gomes et al. (2013) | |
| *Diaporthe terebinthifolii* LGMF907 | KC343217 | KC343943 | Gomes et al. (2013) | |
| *Diaporthe ueckerae* FAU658 | KJ590725 | KJ590746 | Gao et al. (2017) | |
| *Diaporthe yunnanensis* CGMCC 3.18289 | KX986796 | KX999188 | Gao et al. (2017) | |
| *Diaporthe yunnanensis* LC6168 | KX986796.1 | KX999188.1 | Gomes et al. (2013) | |
| *Diaporthella corylina* CBS 121124*** | KC343004 | KC343730 | Gomes et al. (2013) | |

*outgroup

**Table S4**. Results of BLAST analysis on GenBank.

| **Endophyte** | **Gene** | **Species with higher similarity** | **GenBank accession number** | **Identity** |
| --- | --- | --- | --- | --- |
| JB224.g1 | ITS | *Colletotrichum* *siamense* | KP703350.1 | 98% |
|  | GPDH | *Colletotrichum siamense* | MN525877.1 | 87.50% |
|  | β-TUB | *Colletotrichum siamense* | MT199868.1 | 100% |
| JB252.g1 | ITS | *Colletotrichum* *siamense* | KP703350.1 | 99% |
|  | GPDH | *Colletotrichum* *siamense* | MK225167.1 | 85.71% |
|  | β-TUB | *Colletotrichum siamense* | MT199868.1 | 100% |
| JB270 | ITS | *Diaporthe masirevicii* | KJ197281.1 | 98% |
|  | EF1α | *Diaporthe phaseolorum* | MT007103.1 | 89.32% |

**Table S5**. Inhibition index (Im%) and competitive interaction (CI) between 40 endophytic fungal isolates and the phytopathogen *Fusarium oxysporum* (FO).

| **Fungal isolates** | **Mycelial growth of FO (cm)*** | **Im%** | **CI**** |
| --- | --- | --- | --- |
| JB252.g1 | 13.42^a^ ± 0.23 | 68.19 | B |
| JB270 | 16.10^a^ ±7.95 | 61.84 | C_A1_ |
| JB224.g1 | 19.32^b^ ± 3.73 | 54.21 | A |
| JB09 | 19.50^b^ ± 1.94 | 53.76 | C_A1_ |
| JB07 | 20.94^c^ ± 5.38 | 50.35 | B |
| JB176 | 21.07^c^ ± 1.20 | 50.05 | B |
| JB22 | 21.61^c^ ± 0.45 | 48.77 | B |
| JB200 | 21.84^c^ ± 2.04 | 48.22 | C_A1_ |
| JB202 | 21.93^c^ ± 1.18 | 48.01 | C_A1_ |
| JB123.g1 | 22.15^c^ ± 0.29 | 47.49 | A |
| JB35 | 22.35^c^ ± 0.19 | 47.01 | B |
| JB44.g1 | 22.62^c^ ± 4.17 | 46.38 | A |
| JB72 | 22.70^c^ ± 1.97 | 46.19 | A |
| JB185 | 22.82^c^ ± 2.10 | 45.91 | A |
| JB196 | 22.82^c^ ± 4.86 | 45.90 | A |
| JB57 | 23.33^c^ ± 1.39 | 44.70 | C_A1_ |
| JB261 | 23.48^c^ ± 2.85 | 44.34 | A |
| JB89 | 23.65^c^ ± 1.15 | 43.94 | A |
| JB55 | 23.76^c^ ± 1.11 | 43.67 | C_A1_ |
| JB27. g1 | 23.92^c^ ± 1.21 | 43.29 | A |
| JB162 | 25.41^c^ ± 0.95 | 39.77 | A |
| JB76 | 25.64^c^ ± 2.29 | 39.22 | A |
| JB100 | 26.14^c^ ± 1.41 | 38.02 | A |
| JB10 | 26.15^c^ ± 3.82 | 38.01 | A |
| JB90 | 26.16^c^ ± 1.34 | 37.99 | A |
| JB101 | 26.54^c^ ± 0.78 | 37.09 | A |
| JB122 | 26.56^c^ ± 0.79 | 37.04 | A |
| JB73 | 26.70^c^ ± 1.22 | 36.70 | A |
| JB118 | 26.93^c^ ± 0.27 | 36.15 | A |
| JB207.g1 | 27.08^c^ ± 2.62 | 35.81 | A |
| JB175 | 28.38^d^ ± 1.36 | 32.71 | A |
| JB99 | 29.29^d^ ± 2.52 | 30.56 | A |
| JB301.g1 | 29.60^d^ ± 3.42 | 29.82 | A |
| JB107 | 30.14^d^ ± 1.60 | 28.55 | A |
| JB268 | 30.23^d^ ± 2.16 | 28.33 | A |
| JB150 | 30.24^d^ ± 1.48 | 28.32 | A |
| JB25 | 30.29^d^ ± 3.74 | 28.20 | A |
| JB206 | 31.37^d^ ± 1.99 | 25.63 | A |
| JB173 | 35.47^e^ ± 0.49 | 15.92 | A |
| FO (control) | 42.18^f^ ± 1.99 | - | - |

*Mean (± standard deviation) of triplicates followed by different letters indicates that the values are significantly different according to the Scott-Knott test (p < 0.05).

**Badalyan rating scale (Badalyan et al. 2002) where A = deadlock with mycelial contact, B = deadlock at a distance, and C_A1_ = partial replacement after initial deadlock with mycelial contact.

**Table S6**. Soil analysis: chemical parameters and macro and micronutrients.

| **Chemical parameters** | | | | | | | | | | | | | | | | | |
| --- | --- | --- | --- | --- | --- | --- | --- | --- | --- | --- | --- | --- | --- | --- | --- | --- | --- |
| pH(H_2_O) | | H+Al* | | | C** | | M.O(%) | | SB* | | | CTC* | | V(%) | | Ca/Mg | |
| 5.8 | | 2.9 | | | 48.0 | | 8.3 | | 12.90 | | | 15.80 | | 81.65 | | 2.4 | |
| **Macronutrients and micronutrients** | | | | | | | | | | | | | | | | | |
| Ca* | Mg* | | K* | P** | | S** | | Na** | | B** | Fe** | | Mn** | | Cu** | | Zn** |
| 5.6 | 2.3 | | 5.0 | 82.0 | | 8.2 | | 12.5 | | 0.4 | 105.0 | | 18.0 | | 2.5 | | 15.0 |

*(cmol), **(mg/dm3).

**Table S7.** Shoot height (SH), radicular system depth (RSD), number of leaves (NL), SPAD (Soil Plant Analysis Development) index, fresh (FM) and dry mass (DM) of the aerial part (_AP_) and roots (_R_) in leaves of 32 DAT tomato plants inoculated and uninoculated (controls) with fungal endophytes and *F. oxysporum* (FO).

|  | **Treatments** | **SH (cm)** | **F-value**  **(F-critical)** | **RSD (cm)** | **F-value**  **(F-critical)** | **NL** | **F-value**  **(F-critical)** | **SPAD** | **F-value**  **(F-critical)** |
| --- | --- | --- | --- | --- | --- | --- | --- | --- | --- |
| Inoculation of endophytes | Control | 56.40 ± 6.53 | 1,79 (<3,97) | 21.95 ± 3.27 | 0,03 (<3,97) | 10.90 ± 2.13 | **6,99 (>3,97)** | 30.90 ± 3.17 | 3,25 (<3,97) |
|  | JB224.g1 | 54.82 ± 10.60 |  | 23.38 ± 3.12 |  | 10.10 ± 1.28 |  | **35.78 ± 3.48*** |  |
|  | JB252.g1 | 55.99 ± 3.43 |  | 24.58 ± 4.06 |  | **10.00 ± 1.05^#^** |  | **36.09 ± 3.57*** |  |
|  | JB270 | 56.73 ± 4.63 |  | **27.21 ± 2.81*** |  | 9.700 ± 1.41 |  | **34.76 ± 2.49*** |  |
| Inoculation of endophytes and FO | FO | 55.54 ± 6.54 |  | 22.02 ± 3.07 |  | 9.40 ± 1.57 |  | 33.64 ± 3.49 |  |
|  | JB224.g1 + FO | 58.93 ± 3.77 |  | 25.38 ± 2.95 |  | 9.80 ± 1.61 |  | **36.99 ± 3.67*** |  |
|  | JB252.g1 + FO | 55.24 ± 5.90 |  | 23.46 ± 3.20 |  | **9.00 ± 0.94** |  | **35.97 ± 3.29*** |  |
|  | JB270 + FO | 58.54±3.80 |  | **24.81 ± 3.47*** |  | 9.10 ± 1.10 |  | **35.16 ± 3.06*** |  |
| F-value (F-critical) | | 0,57 (<2,73) |  | **3,95 (>2,73)** |  | 1,24 (<2,73) |  | **12,04 (>2,73)** |  |
|  | **Treatments** | **FM_AP_** | **F-value**  **(F-critical)** | **DM_AP_** | **F-value**  **(F-critical)** | **FM_R_** | **F-value**  **(F-critical)** | **DM_R_** | **F-value**  **(F-critical)** |
| Inoculation of endophytes | Control | 37.21 ± 4.93 | **15,48 (>3,97)** | 13.57 ± 0.62 | **23,75 (>3,97)** | 15.09 ± 2.49 | **8,23 (>3,97)** | 9.67 ± 1.46 | 0,01 (<3,97) |
|  | JB224.g1 | 37.35 ± 5.12 |  | **13.72 ± 0.61^#^** |  | **15.97 ± 2.40^#^** |  | **10.60 ± 0.25*** |  |
|  | JB252.g1 | **38.28 ± 4.49^#^** |  | **13.84 ± 0.60^#^** |  | 15.66 ± 2.35 |  | **10.57 ± 0.16*** |  |
|  | JB270 | **36.98 ± 2.94^#^** |  | **13.74 ± 0.39^#^** |  | 13.82 ± 2.30 |  | **10.49 ± 0.12*** |  |
| Inoculation of endophytes and FO | FO | 35.67 ± 3.72 |  | 13.46 ± 0.59 |  | 13.94 ± 2.80 |  | 10.42 ± 0.24 |  |
|  | JB224.g1 + FO | 34.51 ± 3.43 |  | **13.18 ± 0.38** |  | **13.84 ± 2.00** |  | **10.46 ± 0.11*** |  |
|  | JB252.g1 + FO | **32.54 ± 4.42** |  | **12.94 ± 0.53** |  | 14.12 ± 1.79 |  | **10.48 ± 0.14*** |  |
|  | JB270 + FO | **33.04 ± 3.19** |  | **12.98 ± 0.42** |  | 12.86 ± 1.23 |  | 10.29 ± 0.17 |  |
| F-value (F-critical) | | 0,58 (<2,73) |  | 0,35 (<2,73) |  | 2,25 (<2,73) |  | **3,32 (>2,73)** |  |

Means (± standard deviation) are analyzed by two-way ANOVA (p<0.05), using as factors **1**) the presence of endophytes (i.e.: the mean values of JB224.g1 and JB224.g1+FO compared to Control) and **2**) presence of FO *vs*. plants with/without endophytes (i.e.: JB224.g1 *vs*. JB224.g1+FO). * shows the treatments with significant differences compared to control using the factor **1**. # treatments with significant differences to factor **2**. DAT = days after transplanting. Endophytes = *Colletotrichum* *siamense* (codes JB224.g1 and JB252.g1) and *Diaporthe masirevicii* (JB270).


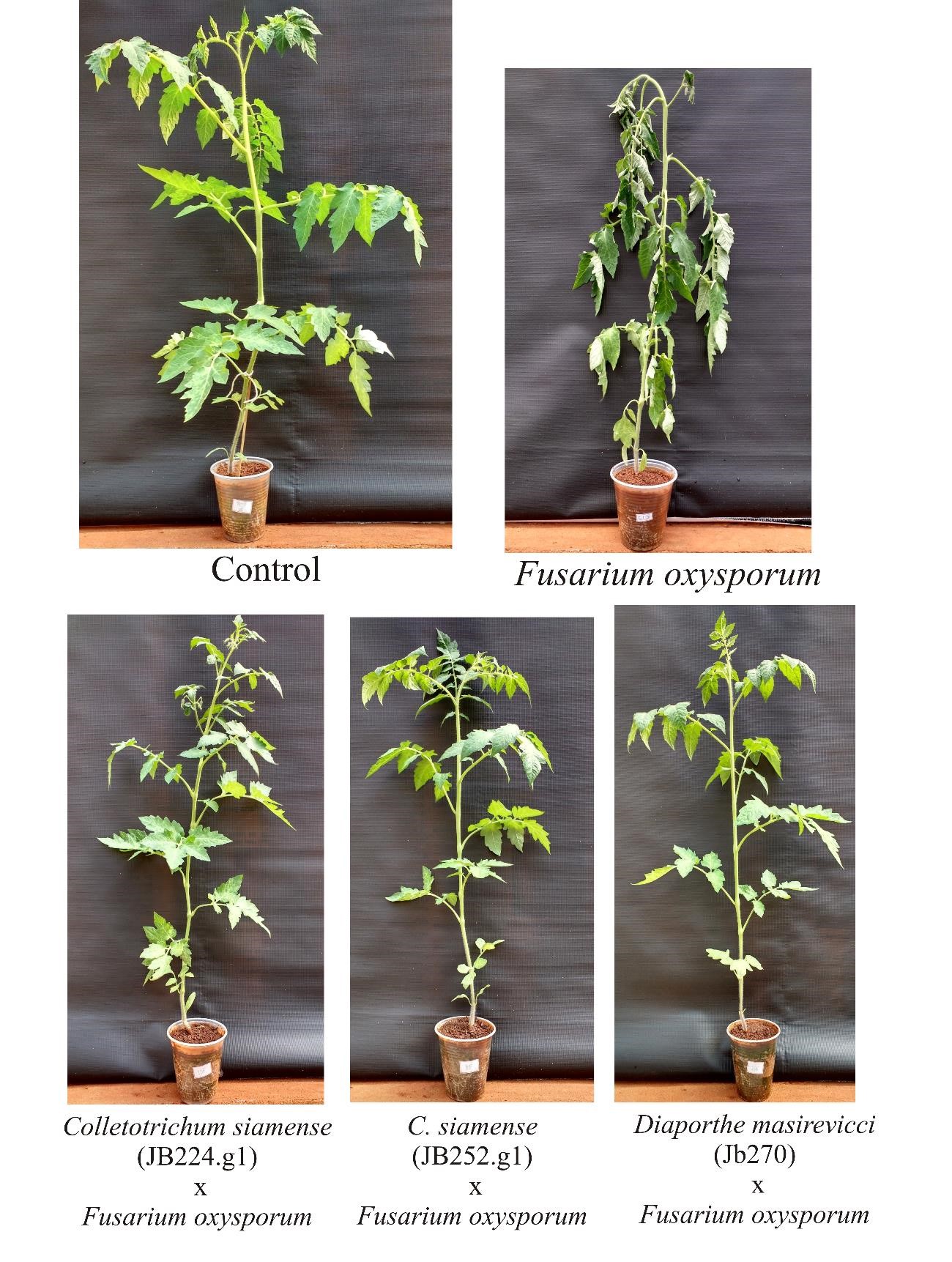


**Figure S1.** Protective effect against phytopathogen *Fusarium oxysporum* observed in tomato plants inoculated with endophytic strains.

**References**

Badalyan SM, Innocenti G, Garibyan NG. 2002. Antagonistic activity of xylotrophic mushrooms against pathogenic fungi of cereals in dual culture. Phytopathol Mediterr. 41:220-225.

Berbee ML, Pirseyedi M, Hubbard S. 1999. *Cochliobolus hylogenetics* and the origin of known, highly virulent pathogens, inferred from ITS and glyceraldehyde-3-phosphate dehydrogenase gene sequences. Mycologia. 91(6):964-977.

Carbone I, Kohn LM. 1999. A method for designing primer sets for speciation studies in filamentous ascomycetes. Mycologia. 91(3):553-556.

Crous PW, Schumacher RK, Wingfield MJ, Akulov A, Denman S, Roux J, Braun U, Burgess TI, Carnegie AJ, Váczy KZ, Guatimosim E, Schwartsburd PB, Barreto RW, Hernández-Restrepo M, Lombard L, Groenewald JZ. 2018. New and interesting fungi. 1. Fungal Syst Evol. 1:169-216.

Damm U, Cannon PF, Woudenberg JH, Johnston PR, Weir BS, Tan YP, Shivas RG, Crous PW. 2012. The *Colletotrichum* boninense species complex. Stud. Mycol. 2012;73(1):1-36.

Damm U, Sato T, Alizadeh A, Groenewald JZ, Crous PW. 2019. The *Colletotrichum dracaenophilum*, *C. magnum* and *C. orchidearum* species complexes. Stud Mycol. 92:1-46.

Fu M, Crous PW, Bai Q, Zhang PF, Xiang J, Guo YS, Zhao FF, Yang MM, Hong N, Xu WX, Wang GP. 2019. *Colletotrichum* species associated with anthracnose of *Pyrus* spp. in China. Persoonia. 42:1-35.

Gao Y, Liu F, Duan W, Crous PW, Cai L. 2017. *Diaporthe* is paraphyletic. IMA Fungus. 8:153-187.

Glass NL, Donaldson GC. Development of primer sets designed for use with the PCR to amplify conserved genes from filamentous ascomycetes. Appl Environ Microbiol. 61(4):1323-1330

Gomes RR, Glienke C, Videira SI, Lombard L, Groenewald JZ, Crous PW. 2013. *Diaporthe*: a genus of endophytic, saprobic and plant pathogenic fungi. Persoonia. 31:1-41.

Liu F, Cai L, Crous PW, Damm U. 2014. The *Colletotrichum* *gigasporum* species complex. Persoonia. 33:83-97.

O'Donnell K, Cigelnik E. 1997. Two divergent intragenomic rDNA ITS2 types within a monophyletic lineage of the fungus *Fusarium* are nonorthologous. Mol Phylogenet Evol. 7(1):103-16.

Thompson SM, Tan YP, Young AJ, Neate SM, Aitken EA, Shivas RG. 2011. Stem cankers on sunflower (*Helianthus annuus*) in Australia reveal a complex of pathogenic *Diaporthe* (*Phomopsis*) species. Persoonia. 27:80-89.

Thompson SM, Tan YP, Shivas RG, Neate SM, Morin L, Bissett A, Aitken EA. 2015. Green and brown bridges between weeds and crops reveal novel *Diaporthe* species in Australia. Persoonia. 35:39-49.

White Jr JF, Marrow AC, Jones GM. 1990. Endophyte-host associations in forage grasses. XII. A fungal endophyte of *Trichachne* *insularis* belonging to *Pseudocercosporella*. Mycologia. 82(2):218-226.
